# Q-Factor Enhancement of Thin-Film Piezoelectric-on-Silicon MEMS Resonator by Phononic Crystal-Reflector Composite Structure

**DOI:** 10.3390/mi11121130

**Published:** 2020-12-20

**Authors:** Jiacheng Liu, Temesgen Bailie Workie, Ting Wu, Zhaohui Wu, Keyuan Gong, Jingfu Bao, Ken-ya Hashimoto

**Affiliations:** 1 School of Electronic Science and Engineering, University of Electronic Science and Technology of China, Chengdu 611731, China; wtbailie@std.uestc.edu.cn (T.B.W.); twu@std.uestc.edu.cn (T.W.); zhwu@std.uestc.edu.cn (Z.W.); 201921020801@std.uestc.edu.cn (K.G.); k.hashimoto@faculty.chiba-u.jp (K.-y.H.); 2 Department of Electrical and Electronic Engineering, Chiba University, Chiba 263-8522, Japan

**Keywords:** MEMS resonator, Q-factor, phononic crystals, acoustic reflector

## Abstract

Thin-film piezoelectric-on-silicon (TPoS) microelectromechanical (MEMS) resonators are required to have high Q-factor to offer satisfactory results in their application areas, such as oscillator, filter, and sensors. This paper proposed a phononic crystal (PnC)-reflector composite structure to improve the Q factor of TPoS resonators. A one-dimensional phononic crystal is designed and deployed on the tether aiming to suppress the acoustic leakage loss as the acoustic wave with frequency in the range of the PnC is not able to propagate through it, and a reflector is fixed on the anchoring boundaries to reflect the acoustic wave that lefts from the effect of the PnC. Several 10 MHz TPoS resonators are fabricated and tested from which the Q-factor of the proposed 10 MHz TPoS resonator which has PnC-reflector composite structure on the tether and anchoring boundaries achieved offers a loaded Q-factor of 4682 which is about a threefold improvement compared to that of the conventional resonator which is about 1570.

## 1. Introduction

With the development trend of miniaturization of electronic equipment, microelectromechanical (MEMS) resonators has shown promising prospects in the field of sensing and wireless communication systems for their property of smaller size, lower power, and higher integration compared to the traditional electrical resonators. However, the quality factor of MEMS resonators with piezoelectric transduction is usually relatively low, which severely limits its practical applications in many fields, such as low phase noise oscillators, high sensitivity sensors, and narrowband filters [1,2,3,4]. For this cause, studying *Q*-factor enhancement strategy for thin film piezoelectric on silicon (TPoS) resonators has paramount importance to promote the practical application of piezoelectric MEMS resonators in many fields. Unlike its quality factor, the effective electromechanical coupling coefficient ($K_{eff}{}^{2}$) of TPoS structure is relatively high and can also achieve single-chip multi-frequency [5,6,7]. As it is well known, the Q-factor is defined as the ratio of the energy stored in the resonator to the energy dissipated for each electromechanical conversion cycle:

(1) $$Q-factor=2\pi \frac{E_{stored}}{E_{dissipated}}$$

where $E_{stored}$ is the vibration energy stored in the resonator and $E_{dissipated}$ denotes the energy dissipated per cycle of vibration, respectively. It is clear that to obtain a higher Q-factor, it requires less energy dissipation. In the last few decades, scholars have been studying the energy loss mechanism of MEMS resonators from which most relevant loss mechanisms in piezoelectric resonators are the anchor loss [8], interface loss, thermoelastic damping (TED), material damping, and other unknown losses. However, it has been found that the anchor loss accounts for a larger proportion of the various energy losses of the resonator. In this work too, the anchor loss is assumed to be the main contributor of various energy dissipation sources, so the total quality factor increases with a higher $Q_{anchor}$ [9,10]. The anchor loss is caused by the mechanical deformation of the resonator during operation, which generates acoustic waves that propagate outward through the tether to the anchoring substrate. The energy carried by the acoustic waves leaky cannot be used by electromechanical conversion in the next cycle. For this reason, different loss reduction schemes were proposed, which can be summarized into two categories: One is to optimize the structure of the resonator itself, such as protrusions on the side edges of the resonator body, etched stress relief holes and anchor points on the resonator body. The other one is the introduction of additional structures around the resonator, such as suspended frames for energy decoupling [11,12,13,14,15,16,17,18,19,20,21,22,23,24,25]. In this study, the conjunction of 1D-phononic crystals (PnCs) on the tether and reflectors on the anchoring boundaries is used to reduce the anchor loss of TPoS resonators. Among them, the phononic crystals structure exhibits a good effect, and the method only needs to design the band gap that includes the resonance frequency, which has a wide range of applications. Moreover, the acoustic reflector is designed with a radius of quarter wavelength for better performance.

## 2. Phononic Crystal Design

**Figure 1:**
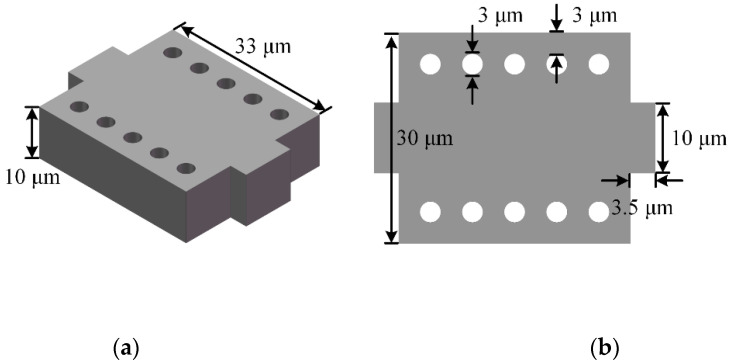
The structure of one-dimensional phononic crystal (PnC). (**a**) 3D schematic view of the proposed PnC configuration; (**b**) Top view of the PnC.

Figure 1 shows the structure of one-dimensional PnC proposed in this paper. Thickness of the PnC must be consistent with the tether as it is intended to be placed on the tether. The holes on the rectangular block are called scatterers, their shape and size affect the band gap of the PnC. The length and width of the PnC are called lattice constants, which also affect the position of the band gap center frequency and the width of the band gap frequency range [26].

Since the one-dimensional PnC has periodicity only in one direction, Bloch-floquent boundary condition is imposed on the wave vector k along the path from point Γ to point X in the first irreducible brillouin zone (IBZ).

**Figure 2:**
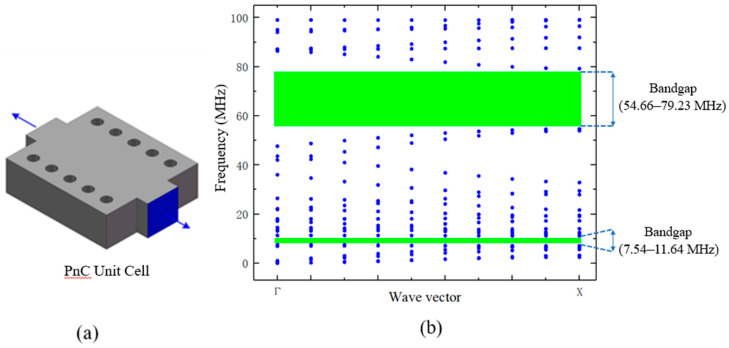
Band gap simulation results of PnC units. (**a**)the blue surface and arrows indicate the position and direction of the applied Bloch boundary conditions; (**b**)the band gap simulation results of the PnC.

The blue surface and arrow in Figure 2 indicate the position and direction of the Bloch boundary conditions applied for the simulation. The dispersion relation of the proposed 1D-PnC is computed using finite element method (FEM) resulting in a complete acoustic bandgap of about 4 MHz (7.54 MHz to 11.64 MHz). The band gap structure of a phononic crystal is closely dependent on the geometric parameters of a single phononic crystal unit. Its size not only determines the existence of the band gap, but also affects the width of the band gap [27]. In addition, the PnCs are usually designed with some special geometric shapes and relatively small in size, so accuracy errors are prone to occur during processing, causing the actual size to be different from the setting during simulation, thereby changing the band gap of the phononic crystal [28,29].

For this reason, several simulations are done to verify the changing trend of the band gap of the phononic crystal with different radius of the circular hole. In this regard, the radius of the circular hole is swept from 0.5 μm to 2.5 μm, while keeping other structural parameters unchanged. The simulated changing trend of the center frequency and width of the phononic crystal bandgap is shown in Table 1 from which it could be observed that the radius of the circular hole affects the center frequency and width of the band gap. The center frequency shows a downward trend from which it sharply drops when the radius increases more than 1.5 μm, while the band gap width has a decreasing trend for the radius less than 1.5 μm and then increased with the increase in the radius. The optimum value can be taken as 1.5 μm considering changing trend of band gap center frequency and width changes at this point. The main reason is that as the radius of the hole increases, the distance between two adjacent holes is too small. For example, for radius of 2.5 μm, the distance between the two circular holes is only 1 μm, and the air in the circular hole and the material between the two circular holes are used as high and low acoustic impedance materials, respectively. As a result, the periodicity will be affected. In addition, when determining the size of the circular hole, the limitations of the processing technology must be considered. Although reducing the radius can increase the band gap width, too small radius may change the shape and size of the scatterer during the processing [26]. After this all comprehensive consideration, a radius of 1.5 μm is finally selected as an optimum value.

**Figure 3:**
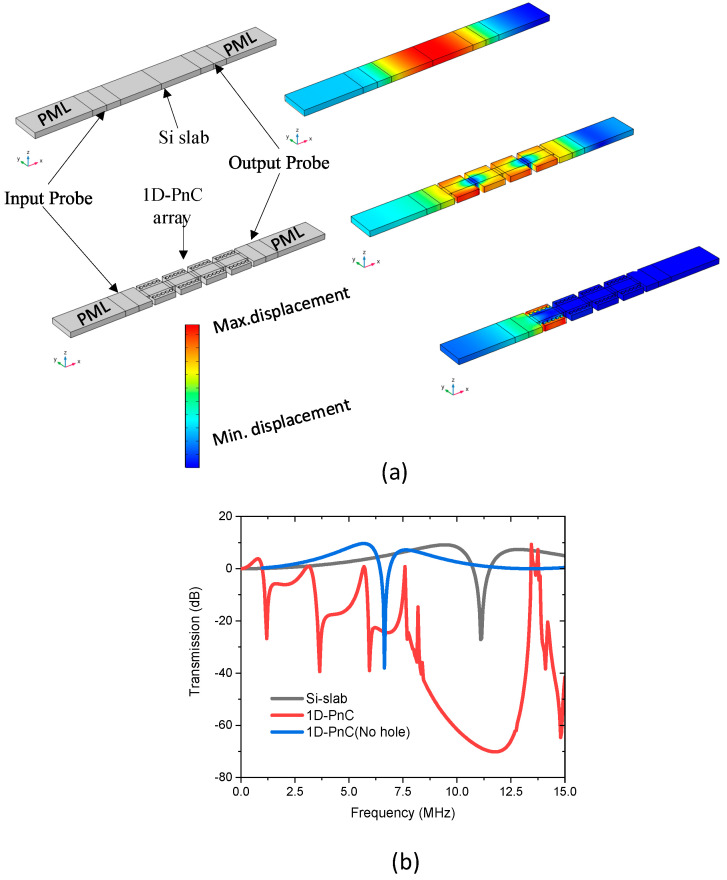
Illustration of transmission characteristics of the proposed 1D-PnC (**a**) delay lines set up to test the transmission of 1D-PnC taking the Si-slab based delay line and a 1D-PnC without hole as a control device. The mode shapes are taken at 10 MHz; (**b**) Transmission (dB) of the delay line made-up of 1D-PnC with and without scatterer holes in comparison with the Control Si slab based delay line.

Furthermore, transmission characteristics of the PnC is simulated and compared with that of a silicon bar used as a transmission medium by using the setup given Figure 3a. In order to reduce the effect of interference by the reflected waves, both sides of the delay line are set to be perfectly matched layers (PML). Prescribed displacement is applied on the input probe and the output probe is set to measure the transmitted response. The transmission characteristics is investigated using transmission parameter which is the measure of transmitted waves from input port to output port ($S_{21}$) in decibels, is expressed as

(2) $$\;T=S_{21}(\mathrm{dB})=10log_{10}\left(\frac{P_{0}}{P_{i}}\right)$$

where $P_{0}$ is output power and $P_{i}$ is input power. Figure 3b reveals a very large drop in the transmission(dB) of the delay line made up of 1D-PnC which shows its tendency in inhibiting the propagation of acoustic waves with frequencies in the bandgap range.

## 3. Resonator Design and Analysis

**Figure 4:**
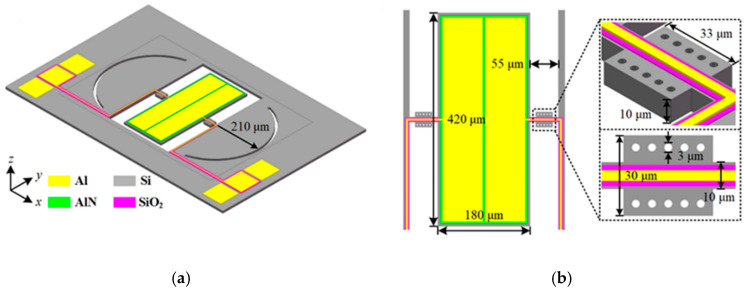
Illustration of the proposed aluminum nitride-on-silicon (AlN-on-Si) microelectromechanical (MEMS) resonators. (**a**) 3D schematic view of the proposed configuration that integrates PnC-reflector composite structure; (**b**) The top view and the enlarged view of the PnC structure at the tether.

The TPoS resonator with PnC-reflector Composite Structure designed in this paper is shown in Figure 4. The figure omits the silicon dioxide layer in the silicon on insulator (SOI) substrate and the back-substrate silicon. The wave equation for a resonator vibrating along its width(*y*-axis) in one space dimension can be written as

(3) $$\rho \frac{\partial^{2}u}{\partial t^{2}}=E_{p}\frac{\partial^{2}u}{\partial y^{2}}$$

where ρ and *Ep* are the equivalent density and Young’s modulus of the material stack forming the bar respectively, *u* is the displacement, *t* is time, and *y* is the direction of vibration. The general solution for Equation (20) can be taken as

(4) $$\;\;\;u\left(y,t\right)=\left[\mathrm{asin}\left(\beta y\right)+bcos\left(\beta y\right)\right]e^{j\omega_{0}t}$$

where βy is the wave vector component in the *y*-axis. Considering a rectangular plate of width $W_{r}$ and applying the boundary conditions that there is no stress and no stress gradient at the free lateral boundaries of the plate (*y* = 0 and *y* =$\;W_{r}$), the fundamental mode of the vibration of the structure can be derived as

(5) $$f_{r}=\frac{n}{2W_{r}}\sqrt{\frac{E_{p}}{\rho }}$$

From Newton–Laplace equation, the acoustic velocity, v in the resonator could be approximated by $\sqrt{\frac{E_{p}}{\rho }}$ [30,31,32,33,34]:

(6) $$f_{r}=\frac{nv}{2W_{r}}$$

where *v* is the acoustic velocity of the resonator,$\;W_{r}$ is the width of the resonator, and *n* is the mode number of the respective harmonic, which is set as one in this work. The resonator is designed to vibrate in symmetrical width extension (WE) mode offering a resonance frequency of 10 MHz. The resonating body of the resonator is a rectangular plate with width ($w_{r}$) = 420 µm, length ($\;L_{r}$) = 180 μm. The plate is actuated through the input electrodes by the inverse piezoelectric effect, while the output electrodes are used to readout the output current converted from the plate vibration through the direct piezoelectric effect. The tether width and perfectly matched layer width (PML) were tuned to be 10 μm and 3 times wavelength(λ) respectively using FEM. The radius of the reflector is designed to be a quarter of the wavelength, 210 μm, of the acoustic wave propagated in the resonator [27]. The resonant body comprises a thin-film piezoelectric layer (0.5 μm thick aluminum nitride, AlN) sandwiched by the upper metallic electrodes (1 μm thick aluminum, Al) and the bottom substrate (10 μm thick silicon, Si). An isotropic silicon with orientation:<110>, for a higher phase velocity [35], is used. The various elastic coefficients are shown in Table 2 [36].

## 4. Fabrication and Measurement

**Figure 5:**
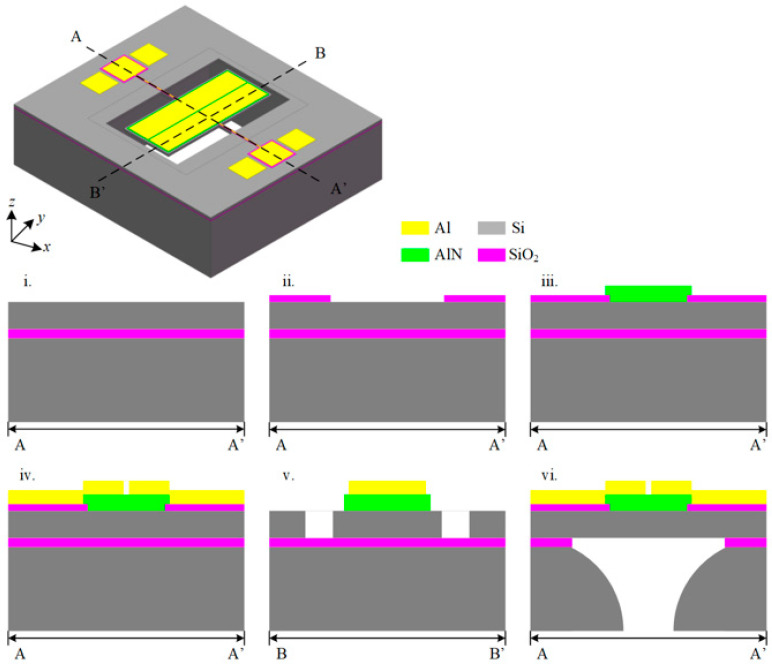
Process flow of thin-film piezoelectric-on-silicon (TPoS) resonator. (**i**) silicon on insulator (SOI) substrate top silicon doping; (**ii**) Thermal oxidation generates insulating layer; (**iii**) Generate piezoelectric film; (**iv**) Generate metal electrodes; (**v**) Top etch; (vi) Back etching.

The TPoS resonators designed in this article are all based on MEMSCAP’s AlN-on-SOI standard process, where by the <110> direction of the wafer is aligned with the *y*-axis direction of the resonator. As shown in Figure 5, the fabrication process of the TPoS resonators follows the following steps:

Step 1. Select the SOI wafer with the <100> crystal orientation, and deposit a layer of Phosphosilicate glass (PSG) on the upper surface of the wafer after polishing. Next, the wafer was annealed in Argon at 1050 °C for one hour, so that PSG was doped onto the top silicon to form a conductive layer, which served as the ground electrode of the entire device. Then the remaining PSG layer is removed by wet etching;

Step 2. The top silicon thermally oxidized to generate a 200 nm thick silicon dioxide layer, and then wet etching is used to pattern the silicon dioxide layer to obtain an insulating layer between the electrode and the ground;

Step 3. A 500 nm thick aluminum nitride film is deposited on the wafer by reactive sputtering, and the designed piezoelectric layer size is obtained by wet etching;

Step 4. Using electron beam evaporation technology 1 μm thick aluminum is deposited and 0.02 μm thick chromium, and the electrodes were patterned through a stripping process and the metal electrodes and wiring of the resonator are obtained;

Step 5. Hard mask deep reactive ion etching (DRIE) is used to etch the top silicon of SOI to the silicon dioxide layer of SOI and support beam structure, mirror structure and phononic crystal structure are obtained;

Step 6. Polyimide is applied as a protective layer on the top of the device to protect the top structure, and then reactive ion etching (RIE) is used to etch the bottom oxide layer for the first time, and then DRIE used to etch from the bottom of the wafer to the second part of the SOI. The silicon oxide layer is then etched with hydrofluoric acid to remove the silicon dioxide layer, so that the resonator body forms a suspension structure. After releasing the top protective layer, the designed TPoS resonator is obtained.

**Figure 6:**
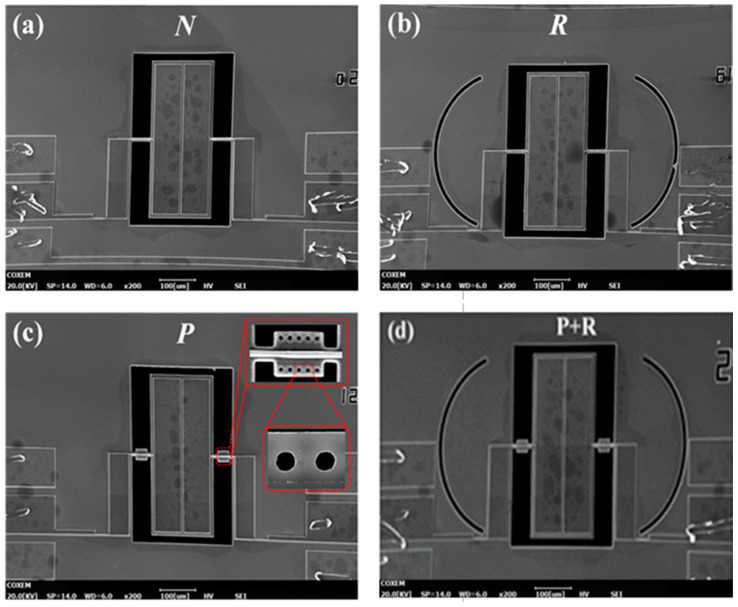
SEM photo of TPoS resonator. (**a**) Conventional structure; (**b**) Resonator with quarter wavelength reflector structure; (**c**) Resonator with 1D-PnC on the supporting tether (**d**) Resonator with 1D PnC-Reflector Composite Structure.

A series of four TPoS resonators with different structures are designed and processed to verify whether the proposed composite structure of phononic crystal and acoustic reflectors can effectively enhance the Q-factor by reducing the anchor loss of the resonator, as shown in Figure 6. The resonant structure of the four TPoS resonators are completely the same except the different additional structures on the supporting structures to suppress anchor loss, and they are all designed to resonate at a resonance frequency of 10 MHz lateral vibration mode.

In Figure 6, (a) is a conventional rectangular plate laterally vibrating resonator, named N; (b) is a resonator with acoustic reflector structure added to the anchoring boundaries, named R, where the reflector structure is used as proposed in [26]. This is only used as a control group to compare the anchor loss suppression effect of a 1D-PnC deployed on the tether. (c) is 1D-PnC deployed on the tether of resonator, named P, (d) is a resonator with a reflector and a 1D-PnC composite structure deployed on the anchoring boundaries and tethers respectively, named R+P. The combination of the two techniques can help the resonator to have reduced anchor loss than that of the techniques used independently as the reflectors can prevent the propagation of acoustic waves leaked from the phononic crystal.

**Figure 7:**
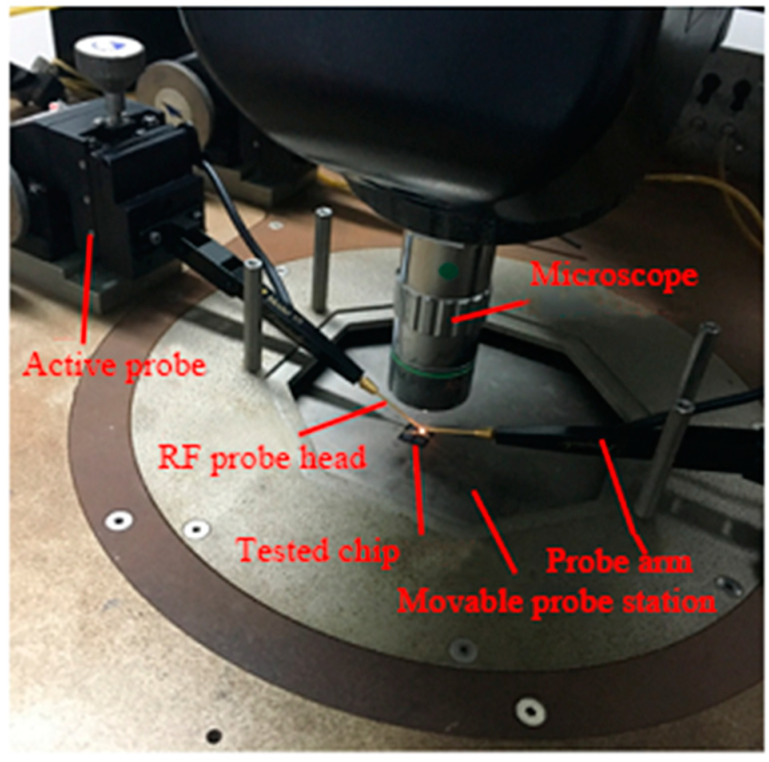
Resonator testing platform.

**Figure 8:**
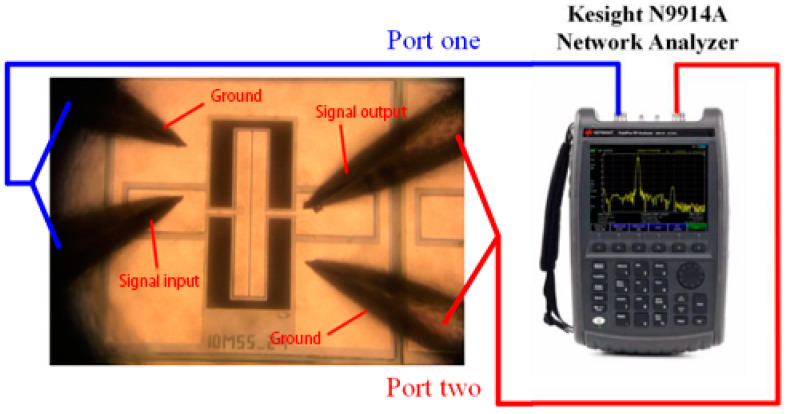
Schematic diagram of the measurement platform.

The electrical performance of the resonator obtained after fabrication is tested. The dust in the air, changes in ambient temperature and humidity should be taken in to considerations during measurement and testing as it will affect the output characteristics of the resonator as the size of the resonator is in the micron level, and the vibration amplitude during operation is usually only tens of nanometers. Due to the aforementioned reasons, the processed resonator is generally packaged to protect the stability of the device output, and for the resonator that is not packaged, the test work must be carried out in a clean room. There is no need for vacuum packaging as TPoS resonator is a piezoelectric resonator for which electromechanical conversion is through a piezoelectric film. Moreover, air is a natural high acoustic impedance material, and its acoustic impedance is much greater than the material of the resonator itself. The acoustic impedance mismatch between the two can effectively prevent the energy in the resonator from leaking into the air. Therefore, even the bare chip test will not have a large impact on its Q-factor. The resonators tested in this article are all bare chips as shown in Figure 7. The test environment is an ordinary clean room at room temperature and standard atmospheric pressure. The test platform used is shown in Figure 8.

The equipment used for the test are:

1. Kesight N9914A 4GHz handheld vector network analyzer
2. Cascade EPS150RF Video Probe Station
3. GGB Picoprobe Model 10 Active Probe Holder
4. GGB Model 30-60-W-2-R-125 RF probe
5. Calibration chip (used for vector network calibration)
6. SMA adapter and coaxial cable

**Figure 9:**
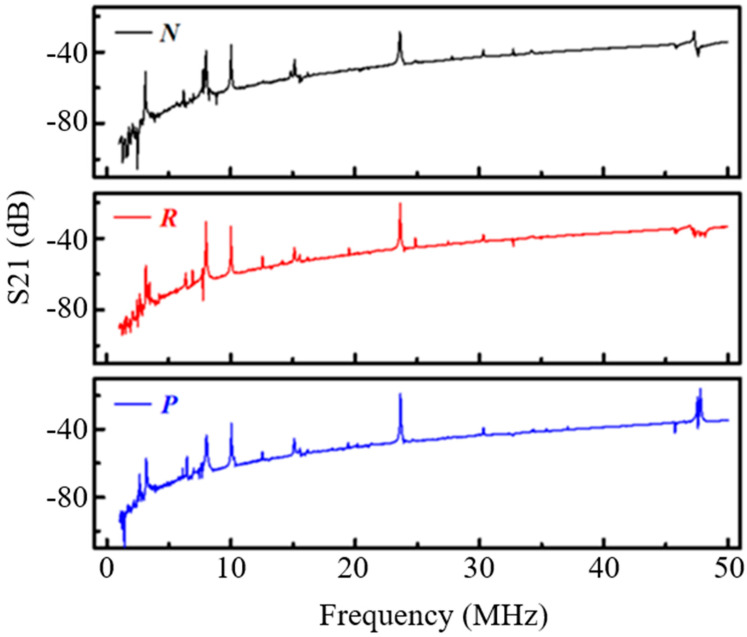
Broadband S21 curve of the resonator, N is the traditional structure, R is the mirror structure, P is the phononic crystal beam structure.

When testing the resonator, first S21 measured with a wider frequency range, as there could be a frequency shift because of the processing accuracy errors. So measuring in a wider frequency range helps to determine the location of the resonant peak, and to observe the remaining multiple resonant peaks produced by the resonator. For this, a wide-band test was conducted first and the results are as shown in Figure 9.

**Figure 10:**
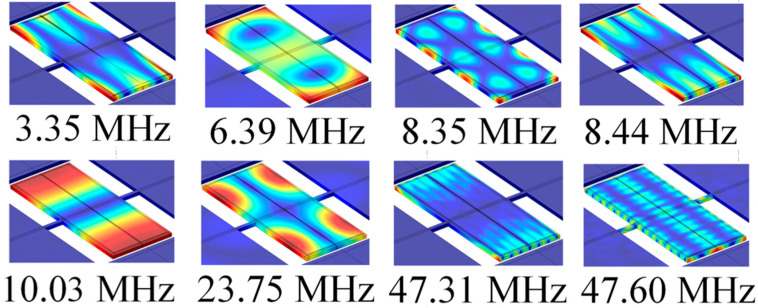
The mode corresponding to each resonance peak in the broadband S21 curve.

From Figure 9, it can be observed that an obvious resonance peak appears at 10.03 MHz, which is almost the same as the resonance frequency obtained by simulation (10.05 MHz), which proves the correctness of the resonator structure design regardless of the small fabrication process error. In addition, there are many resonant peaks besides the resonant frequency. The mode corresponding to each resonant peak are computed through eigen frequency analysis simulation with COMSOL Multiphysics as shown in Figure 10. Although the frequency of these modes is different from the designed basic width extension mode, the frequencies are separated far away, which will not affect the normal operation of the designed mode.

**Figure 11:**
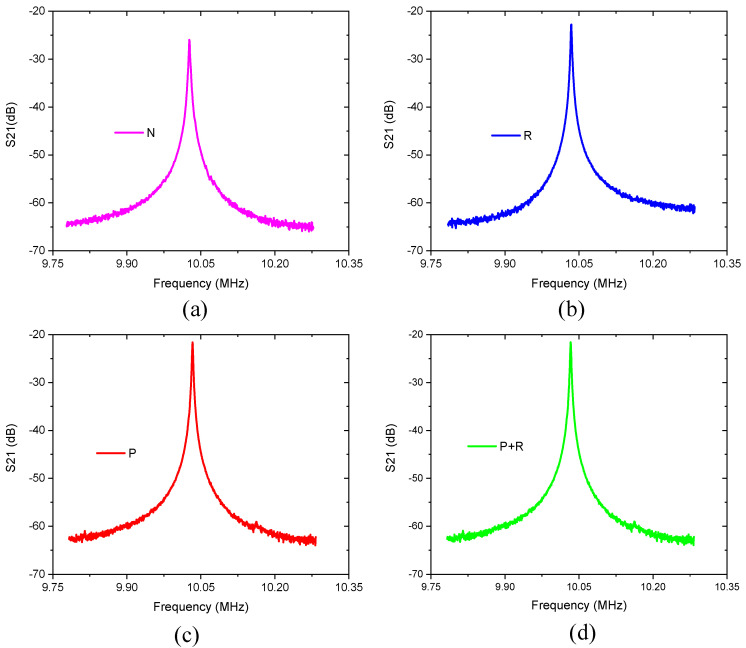
The narrow band S21 curve of the resonator. (**a**) Conventional structure; (**b**) With acoustic reflector on the anchoring boundaries; (**c**) With 1D-PnC on the tether; (**d**) With 1D-PnC and acoustic reflector composite structure.

By changing the measurement bandwidth, a single resonance peak S21, dB curve as shown in Figure 11 can be obtained, and the test results corresponds to the resonator structure in Figure 6. It can be seen from Figure 11 that each S21 curve is very smooth, and there is no spurious mode around the resonance frequency that affects main mode. Through the test, the center frequency ($f_{c}$) and insertion loss (IL) of the resonator can be directly obtained, and the loaded quality factor (Ql) of the resonator can be obtained by calculating the 3dB bandwidth given by the following relation:

(7) $$Q_{l}=\frac{f_{c}}{-3dB\left(\Delta f\right)}$$

where Δ*f* is the 3*dB* band width.

From Figure 11a and Table 3, it can be noticed that the quality factor of the TPoS resonator with the conventional structure is very low, only 1570 compared to one with the acoustic reflectors are added to the anchoring boundaries which is about 1.8 times higher as shown in Figure 11b,c shows the electrical response of the resonator for which the 1D-PnC is deployed on the tethers. Compared with the conventional structure, its Q-factor has been significantly improved (about 2.8 times). Figure 11d shows the electrical response of the resonator which incorporated the two techniques (acoustic reflector and 1D-PnC) showing an improvement in the quality factor with about three times compared to the conventional structure. The experimental results show that the proposed method is very effective in enhancing the Q-factor by suppressing the anchor loss. However, the Q values for other resonant modes were not measured as the study gives due emphasis for the fundamental mode only. In this regard, even though there is no complete band gap at the other modes illustrated with Figure 9, slight improvements were observed in the S21(dB) characteristics of these modes. This could be due to the increase in the acoustic impedance resulting from the deployment of 1D-PnC with scatterer holes.

## 5. Conclusions

A new design approach for improving the quality factor of TPoS MEMS resonators is presented. By using the conjunction of acoustic reflectors and phononic crystals (PnCs), the displacement in the tethers is efficiently suppressed and the energy loss via the tethers is reduced. The acoustic reflectors enables an unloaded Q-factor of up to 2809, showing a 1.8-fold enhancement over the conventional resonator. Whereas the one-dimensional phononic crystal on the tether shows an increased unloaded Q-factor to about 4447 which is about 2.8-fold enhancement. The conjunction of PnCs and reflectors enables the resonator to offer an unloaded Q up to 4682, showing a threefold enhancement over the conventional resonator. The effect of deploying the proposed 1D-PnC on the acoustic impedance change is taken as the next phase of this study.

## Figures and Tables

**Table 1:** Illustration for the effect of Scatterer hole radius on band gap structure of the PnC.

Band Gap Frequency (MHz)	Hole Radius (μm)	Percent Band Gap (%)
10.23	0.5	39.56
9.48	1.0	41.23
9.10	1.5	40.08
9.37	2.0	33.47
10.34	2.5	39.26

**Table 2:** Specific parameters of silicon.

Parameter Name	Value
*Young’s modulus (E)*	$E_{x}=E_{y}=169\;\mathrm{GPa},E_{z}=130\;\mathrm{GPa}$
*Poisson’s ratio (* σ *)*	$\sigma_{\mathrm{xy}}=0.064,\sigma_{\mathrm{yz}}=0.36,\sigma_{\mathrm{zx}}=0.28$
*Shear modulus (G)*	$G_{\mathrm{xy}}=50.9\;\mathrm{GPa},G_{\mathrm{yz}}=G_{\mathrm{zx}}=79.6\;\mathrm{GPa}$
*Density (* ρ *)*	2330 kg/m^3^

**Table 3:** Average values of the measured electrical characteristics of the fabricated resonators.

	*N*	*R*	*P*	*P + R*
Resonant frequency ($f_{0}$), MHz	10.03	10.03	10.03	10.03
Insertion Loss (IL), dB	−27.01	−22.79	−21.64	−21.59
Loaded Quality factor ($Q_{l}$)	1570	2809	4447	4682
